# Assessment of availability, readiness, and challenges for scaling-up hypertension management services at primary healthcare facilities, Central Highland region, Vietnam, 2020

**DOI:** 10.1186/s12875-023-02092-8

**Published:** 2023-07-01

**Authors:** Thang Nghia Hoang, Thuy Phuong Nguyen, Mai Phuong Pham, Hue Kim Le Nguyen, Hieng H, Y Dech Buonya, Tram Dinh Le, Chaisiri Angkurawaranon

**Affiliations:** 1 Tay Nguyen Institute of Hygiene and Epidemiology, Dak Lak, Vietnam; 2 Menzies Institute for Medical Research, University of Tasmania, Hobart, TAS Australia; 3 Institute for Preventive Medicine and Public Health, Hanoi Medical University, Hanoi, Vietnam; 4 Provincial Centers for Diseases Control of Dak Lak, Dak Lak, Vietnam; 5 Provincial Centers for Diseases Control of Dak Nong, Dak Nong, Vietnam; 6 Provincial Centers for Diseases Control of Kon Tum, Kon Tum, Vietnam; 7 Provincial Centers for Diseases Control of Gia Lai, Gai Lai, Vietnam; 8 Department of Family Medicine, Faculty of Medicine, Chiang Mai University, Chiang Mai, Thailand; 9 Global Health and Chronic Conditions Research Group, Chiang Mai University, Chiang Mai, Thailand

**Keywords:** Cardiovascular diseases, Community health services, Community health workers, Hypertension, Non-communicable disease, Preventive medicine, Primary health care

## Abstract

**Introduction:**

Vietnam aims for 95% of commune health stations (CHSs) to have functional hypertension management programs by 2025. However, limited resources may impede the Central Highland region health system from achieving this goal. We assessed the availability and readiness of hypertension management services at CHSs in the Central Highland region and identified challenges to facilitate evidence-based planning.

**Methods:**

We used a mixed-methods cross-sectional design to assess hypertension management services using WHO’s service availability and readiness assessment (SARA) tools in all 579 CHSs in the region, combined with twenty in-depth interviews of hypertension program focal points at communal, district, and provincial levels in all four provinces. We descriptively analyzed quantitative data and thematically analyzed qualitative data.

**Results:**

Hypertension management services were available at 65% of CHSs, and the readiness of the services was 62%. The urban areas had higher availability and readiness indices in most domains (basic amenities, basic equipment, and essential medicines) compared to rural areas, except for staff and training. The qualitative results showed a lack of trained staff and ambiguity in national hypertension treatment guidelines, insufficient essential medicines supply mechanism, and low priority and funding limitations for the hypertension program.

**Conclusion:**

The overall availability and readiness for hypertension diagnosis and management service at CHSs in the Central Highland region were low, reflecting inadequate capacity of the primary healthcare facilities. Some measures to strengthen hypertension programs in the region might include increased financial support, ensuring a sufficient supply of basic medicines, and providing more specific treatment guidelines.

**Supplementary Information:**

The online version contains supplementary material available at 10.1186/s12875-023-02092-8.

## Background

Cardiovascular disease (CVD) was the leading non-communicable disease (NCD) in Vietnam, accounting for 31% (approximately 170,000 cases) of all deaths in 2016, an increase from 25.1% in 2011 [1–4]. The primary CVD risk factor, hypertension (HTN), became prevalent with 18.4% of population between 2002–2015, pooled from three national surveys [5]. However, it is estimated that 30% of people aged 18 to 69 have never had their blood pressure measured by a health care professional and only 14% of patients with hypertension are being managed at health facilities [6]. Additionally, only 12% to 30% of people diagnosed with hypertension have been registered for treatment, and the proportion of treatment adherence was less than 70% [6].

Primary health care centers, namely commune health stations (CHSs), are the first level of care in the Vietnamese health care system, providing both preventive and treatment services [4]. A CHS usually serves 5,000–20,000 people depending on its catchment area [7]. The CHSs could provide effective management and treatment services, but in a national survey of risk factors of NCDs in 2015, they managed approximately 19% of patients with hypertension [8, 9].

The Vietnamese Government has set a national goal of 95% of CHSs implementing hypertension management programs by 2025, but only 12% of all CHSs in Vietnam implemented the hypertension management model in 2018 [10, 11]. For the Central Highland region, one of the most disadvantaged areas in Vietnam, this goal will be difficult to reach. The prevalence of hypertension here was approximately 20% in 2021, in which 11% of patients were managed at CHSs [12–15].

Better understanding of the capacity in hypertension management service provision of CHSs in the Central Highland region will provide baseline evidence for strategic health system planning. We assessed the availability and readiness of hypertension management services at CHSs in the Central Highland region and identified challenges to facilitate evidence-based planning.

## Methods

### Study design

We used a sequential exploratory mixed methods approach and collected both quantitative and qualitative data from January to July 2020, in all four provinces (Dak Lak, Kon Tum, Gia Lai and Dak Nong) of the Central Highland region. Quantitative data from the cross-sectional design provided guidance to select key informants for the qualitative study [16].

### Quantitative method

#### Participants and sampling method

We recruited all CHSs (579/579) within the four provinces of the Central Highland region for a self-administered survey. Participants were CHS staff who were the program manager of the hypertension management program or CHS leaders if the CHS did not have hypertension management program.

#### Operational definitions

Availability of service reflects each facility’s self-reported status from self-administered surveys. Readiness of service reflects the availability of items that were observed and validated at the time of CHSs visit via video calls.

#### Outcomes

We used the adapted the World Health Organization-Service Availability and Readiness Assessment (WHO-SARA) tools to measure the indices for availability and readiness of the hypertension management program at each CHS (Additional file 1). The indices were measured in four domains: basic amenities, staff and training (trained staff and guidelines for diagnosis, treatment, and management of hypertension at CHSs–Guideline No. 5940/2019/QĐ-BYT), basic equipment, and essential medicines and commodities [17].

#### Data collection

To assist data collection in the field, we appointed a focal point to coordinate data collection in each of the four Provincial Centers for Disease Control (PCDCs) and 50 District Health Centers (DHCs). All focal point coordinators were trained on the data collection protocol. The questionnaires were piloted and revised before data collection. District focal points were in charge of following up and collecting self-administered questionnaires and subsequently conducting video interviews with all CHSs. A program manager of the hypertension program at each CHS was selected and invited to fill out the availability questionnaire and then participated in the video interviews following the readiness questionnaire. All questionnaires were collected and sent to the Tay Nguyen Institute for Hygiene and Epidemiology (TIHE) for data entry and analysis.

#### Data analysis

As we collected data from all CHSs within the four provinces of the Central Highland region, only descriptive statistics were used to summarize the characteristics of CHSs. Data were reported as frequencies and percentages or mean and standard deviation (SD). For each item in the questionnaire, facilities were scored one if such item was available/ready at the time of data collection and zero if the item was not available/ready. We calculated the mean score (SD) for each item across all facilities. Availability/readiness index score for each domain (basic amenities, staff and training, basic equipment, and essential medicines and commodities) were calculated by averaging the mean of all items within the domain. Finally, a general availability/readiness index score for the hypertension management program was calculated by averaging the four domain index scores [17]. Data were analyzed using Stata 16.0.

### Qualitative method

#### Data collection

We conducted semi-structured in-depth interviews (IDIs) with key informants (Additional file 2). The interview included four sections. The first section covered general information about the informant, the second section covered information about the health care facilities. The third section was in regard to hypertension management services offered and the last section was about details regarding the implementation of hypertension services. Staff were asked about the four main domains of hypertension management services: basic amenities, staff and training, basic equipment, and essential medicines and commodities. They were encouraged to identify how they could improve services as well as to identify key barriers to implementation.

Based on the results from the quantitative analysis, we purposefully selected two CHSs – one with sufficient level and one with poor level of availability and readiness of hypertension management programs – in each of the four provinces. We invited these key CHS staff for interviews, plus representatives of the NCD program in eight corresponding DHCs and four PCDCs. For CHS staff, questions focused on challenges in implementing day-to-day work on the hypertension management program. For DHCs and PCDCs staff, questions were focused on resources, policies, and financial support for implementing the hypertension management program at the communal level.

#### Data analysis

All interviews were tape-recorded. Each IDI was transcribed word-for-word in the local Vietnamese language and analyzed using NVivo 11.0. Transcripts were coded by two independent team members. We used an `a priory` framework according to the four domains of hypertension services according to the WHO SARA tool for coding and to help identify themes, but also allowed for new themes to emerge. We conducted a thematic analysis across interviews to identify patterns in challenges in implementing a hypertension management program at the communal level and the nature of the factor.

## Results

### Characteristics of the quantitative study population (CHSs)

All 579 CHSs, including 84% (486) in rural areas, provided self-administered questionnaires and participated in video calls. At least one doctor was available in 75% (434/579) of the CHSs (Table 1). One out of ten CHSs had never provided hypertension diagnosis and management services (Table 1).

**Table 1:** Availability staff employed at CHSs in the Central Highland region, Vietnam, 2020

**Staffing category**	**n**^**a**^	**Mean**^**b**^	**% of total CHSs**^**c**^ **(*n* = 579)**
Non-physician clinicians/paramedical professionals	500	1.36 (0.87)	86.4
Generalist (non-specialist) medical doctors	434	0.82 (0.53)	75.0
Nursing professionals	168	0.49 (0.87)	29.0
Midwifery professionals	164	0.36 (0.63)	28.3
Pharmacists	119	0.22 (0.44)	20.6
Specialist medical doctors	40	0.08 (0.3)	6.9
Technician	27	0.05 (0.22)	4.7

*CHSs* Commune health stations

^a^Number of CHSs was calculated by the total number of CHSs having at least one staff

^b^Mean was calculated by the total number of staff divided by the total number of CHSs (579)

^c^Percentage was calculated by the number of CHSs having at least one staff divided by the total number of CHSs (579)

### Hypertension service availability and readiness

Of the self-reported CHSs, 38% had all basic amenities, 75% (434/579) had all essential equipment, 10% (56/579) had well-trained staff, and 5% (31/579) had all essential medicines. Items that CHSs lacked the most were hypertension program guidelines (13%, 76/579), thiazides (15%, 87/579), and beta-blockers (22%, 126/579). Not all CHSs that self-reported an item available had that item ready at the time of video-call validation. The percentage of CHSs with basic amenities readiness was 32% (185/579). No CHSs had all the essential medicines and commodities ready (Table 2).

**Table 2:** Availability and readiness elements of Hypertension management service in CHSs, Central Highland region, Vietnam, 2020

**Domain elements (n = 579)**	**Availability**		**Readiness**	
	**n**	**% of total CHSs**	**n**	**% of total CHSs**
**Basic amenities**				
Environmental disinfectant (e.g., chlorine, alcohol)	579	100	579	100
Computer with MS Office package	567	98	554	96
Hypertension management record books	544	94	538	93
Waste receptacle (pedal bin) with lid and plastic bin liner	531	92	517	89
Disposable latex gloves	508	88	463	80
Landline phone	267	46	253	44
**CHSs with all basic amenities**	**218**	**38**	**185**	**32**
**Staff and training**				
Trained staff for HTN	404	70	404	70
HTN Guidelines	76	13	76	13
**CHSs with both training and guidelines**	**56**	**10**	**56**	**10**
**Basic equipment**				
Thermometer	573	99	570	98
Stethoscope	576	99	574	99
Blood pressure apparatus	573	99	573	99
Adult weighing scale	506	87	506	87
Measuring tape-height board/stadiometer	468	81	469	81
**CHSs with all basic equipment**	**434**	**75**	**434**	**75**
**Essential medicines and commodities**				
Calcium channel blockers	388	67	388	61
ACE inhibitors	334	59	314	54
Beta blockers	126	22	6	1
Thiazides	87	15	72	12
**CHSs with all essential medicines/commodities**	**31**	**5**	**0**	**0**

*CHSs* Commune health stations, *HTN* Hypertension

**Fig. 1:**
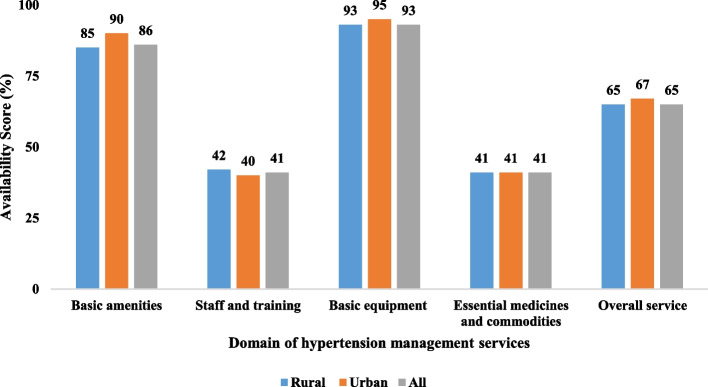
Availability indices of hypertension management program at CHSs in the Central Highland region, Vietnam, 2020. The figure showed availability indices of hypertension management program at commune health stations by service availability domains and commodity types. The overall service availability score was 65% across 579 Communal Health Stations. The urban areas had higher availability indices in all domains compared to the rural areas, except for staff and training

**Fig. 2:**
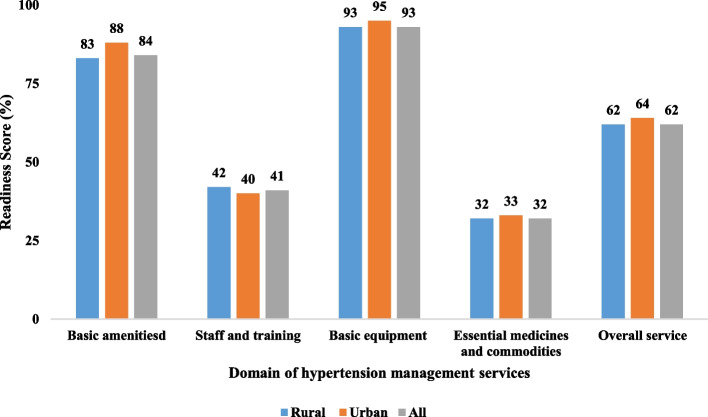
Readiness indices of hypertension management program at CHSs in the Central Highland region, Vietnam, 2020. The figure showed readiness indices of hypertension management program at commune health stations by service readiness domains and commodity types. The overall service readiness score was 62% across 579 Communal Health Stations. The urban areas had higher availability indices in all domains compared to the rural areas, except for staff and training

The overall service availability score was 65% across all CHSs (Fig. 1), slightly higher than the readiness index (62%) (Fig. 2). The essential medicines and commodities domain had both the lowest availability and readiness indices, as well as the largest differences between availability and readiness indices (9 points) (Fig. 1). The urban areas had higher availability indices and readiness indices in all domains compared to the rural areas, except for staff and training. The largest difference in availability and readiness indices between the urban and rural areas was for basic amenities (5 points) (Fig. 2).

### Challenges in provision of hypertension management service at CHSs

We conducted 20 IDIs with health professionals in charge of the hypertension management program at CHSs (*n* = 8), DHCs (*n* = 8), and PCDCs (*n* = 4). All respondents reported experiencing similar challenges to providing the hypertension management services at CHSs. The main challenges included low priority and funding limitations for the hypertension program, staff capacity and management mechanisms, and unstable supply chains of essential medicines and commodities.

### Ambiguity in hypertension treatment guidelines and limited staff capacity

The majority of health professionals (80%, 16/20 participants) indicated that there were multiple national guidelines on hypertension treatment and prevention, causing confusion. They also lacked regular HTN trainings, especially on updated national guidelines.

*“There are many guidelines developed by Ministry of Health or Department of health and many tools has been rolled out for CHSs as well […] CHSs are applying and implementing which guidelines they understand, memorized and simple” – PCDC – Kon Tum Province.*

The high turn-over rate of hypertension focal-points at each level, due to restructuring of Vietnam’s health care system in recent years, could be a contributing factor to the limited capacity of staff. Moreover, with only one medical doctor in charge of all examination and treatment of common diseases at CHSs, their time available to devote to hypertension examination and treatment was not sufficient.

“… *I was assigned for hypertension a few months ago. I still do not clearly understand the hypertension program…”—CHS – Dak Lak province*

### Insufficient essential medicine supply mechanism

The essential medicines for hypertension treatment were not sufficient at CHSs. This theme was identified in 95% (19/20) of the interviews. They were procured and provided through a universal insurance scheme that did not include all essential hypertension drugs. The type of medicines covered by health insurance are different between higher level hospitals and CHSs. As a result, few patients with hypertension received treatment at CHSs. Moreover, patients with hypertension often have comorbidities, such as diabetes or CVDs, requiring integrated care, including laboratory testing, which is not offered by CHSs. Hence, they often bypass CHSs to seek care at higher level facilities or at private providers.

*“ Currently CHSs have three types of basic medicines such as ACE inhibitors, Thiazides, and Beta blockers, which are provided through universal insurance scheme. Although CHSs put in the orders for specific medicines, the medicines we receive [from the insurance scheme] are not the same.” – PCDC – Kon Tum province.*

### Low priorities and funding limitations

All key informants indicated that the hypertension management program lacked funding (100%, 20/20). Basic amenities and equipment at CHSs were provided by DHCs through the Provincial Department of Health (PDH) as part of the essential equipment package for CHSs. No extra equipment was provided by any external funds. The majority of CHSs had one set of blood pressure apparatus, without any backup.

*“… indeed, almost all CHSs in K.B. District is not sufficiently equipped. [We have] only one Blood pressure apparatus, if it is broken, we won’t have anything to replace it.” – DHC – Dak Lak province.*

The budget for hypertension intervention from the government was limited, often being combined with other activities for NCDs, such as mental health and diabetes. Moreover, the hypertension management program was not a priority public health intervention at any level from the communal to the provincial level. The financial support from local health authorities for NCDs was scarce because communicable diseases were viewed as a priority.

*“[…] for hypertension diagnosis, we use our own amenities and equipment, and it is basically enough to provide hypertension service. We did not receive any support from the national program to implement hypertension service …” – CHS – Dak Nong province.*

## Discussion

This study quantified the availability and readiness of hypertension management services at primary health care centers in Vietnam’s Central Highland region. Our study provided the first comprehensive exploration of the current situation of hypertension management programs in this region, as well as identifying the challenges in implementing the program at communal level.

The results provide essential information for developing the strategic plan to strengthen the regional health system. The National guideline recommends staff quantity and composition based on the size of the population and geographical characteristic of the catchment area. According to the guideline, each CHS should have 5 to 10 health staff and at least one medical doctor working full-time or part-time by 2020. However, the results suggest that CHS in the Central Highland region are still understaffed, and many have not been adequately trained. There were slight differences between availability indices (65%) and readiness indices (62%) of hypertension management services in the region. This means approximately two-thirds of the CHSs in the region had the capacity to provide hypertension services in 2020, with urban CHSs somewhat better equipped than rural CHSs. However, meeting the target of having fully functional hypertension services in 95% of CHSs by 2025 remains challenging. We identified three primary areas for improvement, including staff capacity and ambiguity in national hypertension treatment guidelines, insufficient essential medicines supply mechanism, and low priority and funding limitations for the hypertension program.

Compared with results of studies from other regions, the availability of hypertension services in the Central Highland region was lower. A study from Northern Vietnam reported 88% of CHSs had hypertension services available [13, 14], while a study in Ho Chi Minh City (the largest city in Southern Vietnam) showed that 100% of CHSs had hypertension services available [18]. As for service readiness, there were no large-scale studies in Vietnam. Results from single provinces reported both lower (51%) and higher (82%) [15] levels of hypertension service readiness compared to our results (63%). Compared with the results of from other countries, the regional service readiness was both lower and higher than that in other countries. A study from Uganda demonstrated a higher level of service readiness for hypertension management (86%), but with weaknesses in capacity of health staffs and lack of functional equipment [19]. While another study from Ethiopia reported lower levels of service readiness for Hypertension management at primary health care units (29%), influenced by insufficient equipment for screening and diagnostic, insufficient essential medicines, and lack of basic training and guideline for non-communicable diseases [20]. However, results should be interpreted with caution as the discrepancies between studies could be due to study location, sample size or instruments used.

The qualitative results provided context for better understanding and interpretation of our availability and readiness assessment. Most CHSs in our study were reported to experience a lack of trained health staff and low availability of essential medicines and commodities. This was similar with findings from other studies in which the shortage of hypertension services was mainly caused by a lack of basic medicines, national guidelines, and trained health staff [2, 7, 8, 21, 22]. The low level of availability and readiness of trained staff at CHSs has been explained by the lack of training and the high turnover rate due to restructuring of the health system in recent years [23].

Well prepared and standardized guidelines for diagnosis and treatment of hypertension could play a key role in offering consistent hypertension management services [2]. However, IDI respondents noted multiple guidelines with conflicting guidance, which confused health professionals. The inconsistencies included differences in hypertension diagnostic procedures (threshold, and steps of diagnosis) and management practices at CHSs [5].

Insufficient essential medicines readiness hindered providing hypertension service at CHSs. These medicines are provided through a universal insurance scheme and are out of the control of the local health system [5]. Lack of sufficient essential medicines may reduce patient visits at CHSs and overload health facilities at higher levels.

The lack of financial support led to ineffective implementation of hypertension activities, such as training, and behavioral change communication and education. Much of the government’s support for preventive healthcare, more than 60% in 2011 of the national health budgets, was focused on infectious disease [2]. Despite 20 years of Vietnam’s NCD prevention and control strategy, there remains a lack of financial support at the national level. Lack of training also resulted from limited financial support, and insufficient training makes it difficult for providers to implement guidelines, especially at the commune level [2].

Our study has several strengths. We used a mixed methods approach with qualitative data to complement and enhance the interpretation of the quantitative data to comprehensively answer our research questions. We also piloted our questionnaires and had clear quality assurance for the data collection process to assure the accuracy of the data. However, there were limitations in our study. Firstly, the WHO-SARA tool could not capture other aspects of access to health services such as geographical, economic, or quality aspect of the services. Secondly, self-reported questionnaires were used to assess availability, which may have led to both systematic and random errors. Readiness was validated /observed during video-visits rather than in-person visits, and this could have been a source of data collection bias. Thirdly, due to resource constraints, we could not select all CHSs for qualitative interviews. However, by selecting two centers (one high and one low performance) from each of the four provinces, we diversified our sample and were still able achieved data saturation. Lastly, this study only focused on the Central Highland region. Therefore, the results may not be generalizable to the rest of Vietnam, particularly to large metropolitan areas with more advanced socioeconomic status.

## Conclusions

In conclusion, the overall availability and readiness of hypertension management services at CHSs in Central Highland were low, reflecting the low capacity of the primary healthcare facilities. To strengthen the hypertension program in the Central Highland region and to meet the goal of 95% of CHSs providing effective hypertension programs by 2025, the health authorities could increase financial support, ensure adequate types of basic medicines, and issue clear guidelines for the prevention, management, and treatment of hypertension for the CHSs. Public health actions should include: (1) Develop policy on capacity building for local health professionals and human resources retention to maintain a proper workforce at the grassroots level for essential NCD services; (2) The Ministry of Health (MoH) should streamline standardized guidelines on hypertension management at CHSs and provide training on the guideline to all CHSs for consistency; (3) More financial support is needed for the NCD program, not only from the MoH budget line but also from the Provincial Government budget; (4) Improve the essential medicine procurement process by allowing long-term essential medicine procurement instead of quarterly/biannual/annual procurement. The Provincial Department of Health, who oversees conducting competitive bidding to buy essential medicines, then redistributes the medicines to CHSs, should have a long-term plan for procurement to secure a stable supply of essential medicines for their jurisdictions. At the higher level, the MoH and Ministry of Social Welfare need to agree on the list of essential medicines provided through universal insurance scheme to meet the need for hypertension treatment.

## Supplementary Information

**Additional file 1.**

**Additional file 2.**

## Data Availability

Data are available on request due to privacy restrictions. The datasets during and/or analyzed during the current study available from the corresponding author on reasonable request.

## Abbreviations

CVD: Cardiovascular disease
NCD: Non-communicable disease
HTN: Hypertension
CHS: Commune health station
WHO-SARA: World Health Organization-Service Availability and Readiness Assessment
PCDC: Provincial Centers for Disease Control
DHC: District Health Center
TIHE: Tay Nguyen Institute for Hygiene and Epidemiology
SD: Standard deviation
IDI: In-depth interview
PDH: Provincial Department of Health
TEPHINET: Training Programs in Epidemiology and Public Health Interventions Network
