# Supplementary material for: Assessment of availability, readiness, and challenges for scaling-up hypertension management services at primary healthcare facilities, Central Highland region, Vietnam, 2020
Source: BMC Prim Care. 2023 Jul 1;24:138. doi: 10.1186/s12875-023-02092-8 (PMC10315019; doi:10.1186/s12875-023-02092-8)
Supplement: Supplementary file 1 — Additional file 1. [file 12875_2023_2092_MOESM1_ESM.docx]

**Part 1**

**Availability and readiness of hypertension management services questionnaire**

**A – AVAILABILITY QUESTIONNAIRE (Self-administered by health staff at CHS)**

| **Indicator code** | **Number** | **Question** | **Results** | | **Skip** | | | | |  |
| --- | --- | --- | --- | --- | --- | --- | --- | --- | --- | --- |
| **PART 1: GENERAL INFORMATION** | | | | | | | | | | |
|  | 001 | Facility code | \|__\|\|__\|\|__\|\|__\|\|__\| | | (to be filled by district coordinator) | | | | |  |
|  | 002 | Date of interview/response | …./……./…………. | |  | | | | |  |
|  | 003 | Name of facility | ___________________ | |  | | | | |  |
|  |  | Location of facility | ___________________ | |  | | | | |  |
|  |  | District | ___________________ | |  | | | | |  |
|  |  | Province | ___________________ | |  | | | | |  |
|  |  | Urban/Rural | URBAN ............................................................................... 1  RURAL .............................................................................. 2 | | | | | | |  |
| Good day! My name is _____________________. We are here on behalf of Tay Nguyen Institute of Hygiene and Epidemiology conducting a survey of health facilities to assist the government in knowing more about health services in Central Highland, Vietnam. Now I will read a statement explaining the study. Your facility was selected to participate in this study. We will be asking you questions about various services of hypertension management. Information about your facility may be used by the Provincial health department and Ministry of Health, organizations supporting services in your facility, and researchers, for planning hypertension management service improvement or for conducting further studies of non-communicable diseases (NCD) related health services. Neither your name nor that of any other health worker respondents participating in this study will be included in the dataset or in any report; however, there is a small chance that any of these respondents may be identified later. Still, we are asking for your help to ensure that the information we collect is accurate. You may refuse to answer any question or choose to stop the interview at any time. However, we hope you will answer the questions, which will benefit the hypertension management services you provide and the nation. If there are questions for which someone else is the most appropriate person to provide the information, we would appreciate if you introduce us to that person to help us collect that information. At this point, do you have any questions about the study? Do I have your agreement to proceed?  _________________________________________ ______/________/_________________  **INTERVIEWER'S SIGNATURE INDICATING CONSENT OBTAINED DAY MONTH YEAR** | | | | | | | | | | |
| **PART 2: SERVICE AVAILABILITY** | | | | | | | | | | |
| **SECTION 1: STAFFING AND GUIDELINES FOR HYPERTENSION MANAGEMENT** | | | | | |  | | | | |
| A1 | 100 | Please tell me how many staff with each of the following qualifications are currently assigned to, employed by, or seconded to this facility to work on hypertension management program. Please count each staff member only once, on the basis of the highest technical or professional qualification. For doctors, I would also like to know, of the total number, how many are part-time in this facility. | **A) ASSIGNED/ EMPLOYED/ SECONDED (INCLUDING PART TIME)** | | **B) PART TIME** | | | | |  |
|  | 01 | Generalist (non-specialist) medical doctors | \|___\|___\|\|___\| | | \|___\|___\|\|___\| | | | | |  |
|  | 02 | Specialist medical doctors | \|___\|___\|\|___\| | | \|___\|___\|\|___\| | | | | |  |
|  | 03 | Non-physician clinicians/paramedical professionals | \|___\|___\|\|___\| | | \|___\|___\|\|___\| | | | | |  |
|  | 04 | Nursing professionals | \|___\|___\|\|___\| | | \|___\|___\|\|___\| | | | | |  |
|  | 05 | Midwifery professionals | \|___\|___\|\|___\| | | \|___\|___\|\|___\| | | | | |  |
|  | 06 | Pharmacists | \|___\|___\|\|___\| | | \|___\|___\|\|___\| | | | | |  |
|  | 07 | Community health workers | \|___\|___\|\|___\| | | \|___\|___\|\|___\| | | | | |  |
| A1 | 200 | Please list all guidelines on hypertension management available to you | If select yes, please specify the number of guidelines and all the titles of the guidelines that you are aware of, and who enacted the guidelines in the next column for each level. | | | | | | |  |
|  | 01 | National guidelines | YES NO | |  | | | | |  |
|  | 02 | Provincial guidelines | YES NO | |  | | | | |  |
|  | 03 | District guidelines | YES NO | |  | | | | |  |
|  | 04 | Communal guidelines | YES NO | |  | | | | |  |
| **SECTION 2: EQUIPMENT** | | | | | | | | | | |
| A2 | 100 | Please tell me if the following basic equipment and supplies used in the provision of client services are available and functional in this facility today. |  | | | | | |  |  |
|  | 01 | Adult weighing scale | YES (Quantity) _____________ NO | | | | | |  |  |
|  | 02 | Measuring tape-height board/stadiometer | YES (Quantity) _____________ NO | | | | | |  |  |
|  | 03 | Thermometer | YES (Quantity) _____________ NO | | | | | |  |  |
|  | 04 | Stethoscope | YES (Quantity) _____________ NO | | | | | |  |  |
|  | 05 | Blood pressure apparatus (may be digital or manual sphygmomanometer with stethoscope) | YES (Quantity) _____________ NO | | | | | |  |  |
|  | 06 | Clean running water (piped, bucket with tap, or pour pitcher) | YES (Quantity) _____________ NO | | | | | |  |  |
|  | 07 | Hand-washing soap/liquid soap | YES (Quantity) _____________ NO | | | | | |  |  |
|  | 08 | Alcohol based hand rub | YES (Quantity) _____________ NO | | | | | |  |  |
|  | 09 | Disposable latex gloves | YES (Quantity) _____________ NO | | | | | |  |  |
|  | 10 | Waste receptacle (pedal bin) with lid and plastic bin liner | YES (Quantity) _____________ NO | | | | | |  |  |
|  | 11 | Environmental disinfectant (e.g., chlorine, alcohol) | YES (Quantity) _____________ NO | | | | | |  |  |
|  | 12 | Computer with MS Office package | YES (Quantity) _____________ NO | | | | | |  |  |
|  | 13 | Hypertension management record books | YES (Quantity) _____________ NO | | | | | |  |  |
|  | 14 | Landline phone | YES (Quantity) _____________ NO | | | | | |  |  |
| **SECTION 3: MEDICINES, COMMOBIDITIES, AND SUPPLY CHAIN** | | | | | | | | | |  |
| A3 | 100 | Who is the principal person responsible for managing the ordering of medical supplies at this facility? | NURSE ........................................................................1  CLINICAL OFFICER ....................................................2  PHARMACY TECHNICIAN ..........................................3  PHARMACY ASSISTANT ........................................... 4  PHARMACIST...............................................................5  MEDICAL ASSISTANT .............................................. 6  OTHER (SPECIFY)__________________________ 96 | | | | | |  |  |
| A3 | 200 | Which of the following mechanisms is used to determine this facility’s resupply quantities? ASK FOR EACH OF THE BELOW | YES | NO | DON’T KNOW | | | | |  |
|  | 01 | The facility itself | 1 | 2 | 3 | | | | |  |
|  | 02 | A higher-level facility | 1 | 2 | 3 | | | | |  |
|  | 03 | Other (SPECIFY)__________________ | 1 | 2 | 3 | | | | |  |
| A3 | 300 | How are the facility’s resupply quantities determined? | FORMULA (ANY CALCULATION) .........................1  DON’T KNOW ....................................................... 2  OTHER MEANS ................................................... 3 | | | |  | | |  |
| A3 | 400 | What is the main source of your routine pharmaceutical commodity supplies? By this I mean who is the direct supplier to your facility? | NATIONAL MEDICAL STORES ........................... 1  JOINT MEDICAL STORES ................................... 2 NGO/DONORS ..................................................... 3 PRIVATE SOURCES ............................................ 4 OTHER (SPECIFY)_______________________ 96 | | | |  | | |  |
| A3 | 500 | How are your pharmaceutical commodity supplies from the main supplier of your routine pharmaceuticals delivered to this facility? | SUPPLIER DELIVERS TO FACILITY ................... 1 FACILITY MUST ARRANGE DELIVERY TO FACILITY .............................................................. 2  OTHER (SPECIFY)_______________________ 96 | | | |  | | |  |
| A3 | 600 | Who is responsible for transporting products from central medical stores to your facility? | YES | NO |  | | | | |  |
|  | 01 | Local supplier delivers | 1 | 2 |  | | | | |  |
|  | 02 | Higher level delivers | 1 | 2 |  | | | | |  |
|  | 03 | This facility collects | 1 | 2 |  | | | | |  |
|  | 04 | Other (SPECIFY)__________________ ________________________________ | 1 | 2 |  | | | | |  |
| A3 | 700 | For the most recent order, how long did it take between ordering and receiving products? | LESS THAN 2 WEEKS................................................. 1  2 WEEKS TO 1 MONTH .............................................. 2  BETWEEN 1 AND 2 MONTHS .................................... 3  MORE THAN 2 MONTHS ............................................ 4 | | | | |  | |  |
| A3 | 800 | Are any of the following medicines for the management of **hypertension** available (count in tablets) in the facility today? |  | | | | |  | |  |
|  | 01 | Enalapril | YES (Quantity) _____________ NO | | | | |  | |  |
|  | 02 | Enalapril+Hydrochlorothiazid | YES (Quantity) _____________ NO | | | | |  | |  |
|  | 03 | Nifedipin | YES (Quantity) _____________ NO | | | | |  | |  |
|  | 04 | Carvedilol | YES (Quantity) _____________ NO | | | | |  | |  |
|  | 05 | Losartan | YES (Quantity) _____________ NO | | | | |  | |  |
|  | 06 | Losartan + hydroclorothiazid | YES (Quantity) _____________ NO | | | | |  | |  |
|  | 07 | Nicardipine | YES (Quantity) _____________ NO | | | | |  | |  |
| **SECTION 4: INTERVIEWER'S OBSERVATIONS** | | | | | | | | | |  |
| A4 | 100 | INTERVIEW END TIME (use the 24 hour-clock system) | \|___\| \|___\|: \|___\| \|___\| | | | | |  | |  |
| A4 | 200 | RESULT CODES  (LAST VISIT): | COMPLETED ......................................................1 RESPONDENT NOT AVAILABLE ...................... 2 REFUSED ............................................................3 PARTIALLY COMPLETED .................................. 4 OTHER (SPECIFY)_______________________96 | | | | |  | |  |

**B – READINESS QUESTIONNAIRE (Administered by district coordinator)**

| **Indicator code** | **Number** | **Question** | **Results** | | | | | | **Skip** | | | | | | | | | |  |
| --- | --- | --- | --- | --- | --- | --- | --- | --- | --- | --- | --- | --- | --- | --- | --- | --- | --- | --- | --- |
| **PART 1: GENERAL INFORMATION** | | | | | | | | | | | | | | | | | | | |
|  | 001 | Facility code | \|__\|\|__\|\|__\|\|__\|\|__\| | | | | | | (to be filled by district coordinator) | | | | | | | | | |  |
|  | 002 | Date of interview/response | …./……./…………. | | | | | |  | | | | | | | | | |  |
|  | 003 | Name of facility | ___________________ | | | | | |  | | | | | | | | | |  |
|  |  | Location of facility | ___________________ | | | | | |  | | | | | | | | | |  |
|  |  | District | ___________________ | | | | | |  | | | | | | | | | |  |
|  |  | Province | ___________________ | | | | | |  | | | | | | | | | |  |
|  |  | Urban/Rural | URBAN ............................................................................... 1  RURAL .............................................................................. 2 | | | | | | | | | | | | | | | |  |
| Good day! My name is _____________________. We are here on behalf of Tay Nguyen Institute of Hygiene and Epidemiology conducting a survey of health facilities to assist the government in knowing more about health services in Central Highland, Vietnam. Now I will read a statement explaining the study. Your facility was selected to participate in this study. We will be asking you questions about various services of hypertension management. Information about your facility may be used by the Provincial health department and Ministry of Health, organizations supporting services in your facility, and researchers, for planning hypertension management service improvement or for conducting further studies of non-communicable diseases (NCD) related health services. Neither your name nor that of any other health worker respondents participating in this study will be included in the dataset or in any report; however, there is a small chance that any of these respondents may be identified later. Still, we are asking for your help to ensure that the information we collect is accurate. You may refuse to answer any question or choose to stop the interview at any time. However, we hope you will answer the questions, which will benefit the hypertension management services you provide and the nation. If there are questions for which someone else is the most appropriate person to provide the information, we would appreciate if you introduce us to that person to help us collect that information. At this point, do you have any questions about the study? Do I have your agreement to proceed?  _________________________________________ ______/________/_________________  **INTERVIEWER'S SIGNATURE INDICATING CONSENT OBTAINED DAY MONTH YEAR** | | | | | | | | | | | | | | | | | | | |
| **PART 2: SERVICE READINESS** | | | | | | | | | | | | | | | | | | | |
| **SECTION 1: AVAILABLE SERVICES** | | |  | | | | | |  | | | | | | | | | |  |
| R1 | 100 | Does this facility offer diagnosis or management of non-communicable diseases diabetes, hypertension, cardiovascular disease, chronic respiratory disease, or cervical cancer? | YES ............................................................. 1  NO ............................................................. 2 | | | | | | | | | | | | | | | à R2 |  |
|  | ASK TO BE SHOWN THE LOCATION IN THE FACILITY WHERE NON-COMMUNICABLE DISEASE SERVICES ARE PROVIDED. FIND THE PERSON MOST KNOWLEDGEABLE ABOUT NCD SERVICES IN THE FACILITY. INTRODUCE YOURSELF, EXPLAIN THE PURPOSE OF THE SURVEY AND ASK THE FOLLOWING QUESTIONS. | | | | | | | | | | | | | | | | | | |
| R1 | 200 | Do providers in this facility diagnose and/or manage cardiovascular diseases such as hypertension in patients? | YES…………………………………............. 1  NO ............................................................. 2 | | | | | | | | | |  | | | | | |  |
| R1 | 300 | Do you have the national guidelines for the diagnosis and management of hypertension available in this facility today?  **IF AVAILABLE, ASK TO SEE THE DOCUMENT** | YES…………………………………............. 1  NO ............................................................. 2 | | | | | | | | | |  | | | | | |  |
| R1 | 400 | Have you or any provider(s) of services for hypertension management received any training in the diagnosis and management of hypertension in the last two years? | YES…………………………………............. 1  NO ............................................................. 2 | | | | | | | | | |  | | | | | |  |
| **SECTION 2: BASIC EQUIPMENT FOR HYPERTENSION MANAGEMENT PROGRAM** | | |  | | | | | |  | | | | | | | | | |  |
| R2 | 100 | Please tell me if the following basic equipment and supplies used in the provision of client services are available and functional in this facility today.  **ASK TO SEE THE ITEMS** | **A) AVAILABLE** | | | | | | **B) FUNCTIONING** | | | | | | | | | |  |
|  |  |  | YES | NO | | | | | YES | | NO | | | | | | DON’T KNOW | |  |
|  | 01 | Please tell me if the following basic equipment and supplies used in the provision of client services are available and functional in this facility today. | 1 | 2 | | | | | 1 | | 2 | | | | | | 99 | |  |
|  | 02 | Adult weighing scale |  |  | | | | |  | |  | | | | | |  | |  |
|  | 03 | Measuring tape-height board/stadiometer | 1 | 2 | | | | | 1 | | 2 | | | | | | 99 | |  |
|  | 04 | Thermometer | 1 | 2 | | | | | 1 | | 2 | | | | | | 99 | |  |
|  | 05 | Stethoscope | 1 | 2 | | | | | 1 | | 2 | | | | | | 99 | |  |
|  | 06 | Blood pressure apparatus (may be digital or manual sphygmomanometer with stethoscope) | 1 | 2 | | | | | 1 | | 2 | | | | | | 99 | |  |
| **SECTION 2: OTHER EQUIPMENT** | | |  | | | | | | | | | | | | | | |  |  |
| R2 | 200 | Please tell me if the following resources/supplies used for infection control are available in the general outpatient area of this facility today.  **ASK TO SEE THE ITEMS** | **OBSERVED** | | | | | **REPORTED NOT SEEN** | | | | **NOT**  **AVAILABLE** | | | | | | |  |
|  | 01 | Clean running water (piped, bucket with tap, or pour pitcher) | 1 | | | | | 2 | | | | 3 | | | | | | |  |
|  | 02 | Hand-washing soap/liquid soap | 1 | | | | | 2 | | | | 3 | | | | | | |  |
|  | 03 | Alcohol based hand rub | 1 | | | | | 2 | | | | 3 | | | | | | |  |
|  | 04 | Disposable latex gloves | 1 | | | | | 2 | | | | 3 | | | | | | |  |
|  | 05 | Waste receptacle (pedal bin) with lid and plastic bin liner | 1 | | | | | 2 | | | | 3 | | | | | | |  |
|  | 06 | Environmental disinfectant (e.g., chlorine, alcohol) | 1 | | | | | 2 | | | | 3 | | | | | | |  |
|  | 07 | Computer with MS Office package | 1 | | | | | 2 | | | | 3 | | | | | | |  |
|  | 08 | Hypertension management record books | 1 | | | | | 2 | | | | 3 | | | | | | |  |
|  | 09 | Landline phone | 1 | | | | | 2 | | | | 3 | | | | | | |  |
| **SECTION 3: MEDICINES AND COMMODITIES** | | |  | | | | | |  | | | | | | | | | |  |
| R3 | 100 | Does this facility stock medicines and commodities? | YES ............................................................ 1  NO ............................................................. 2 | | | | | | | | | | | àR4 | | | | |  |
|  | ASK TO BE SHOWN THE MAIN LOCATION IN THE FACILITY WHERE MEDICINES AND OTHER SUPPLIES ARE STORED. FIND THE PERSON MOST KNOWLEDGEABLE ABOUT STORAGE AND MANAGEMENT OF MEDICINES AND SUPPLIES IN THE FACILITY. INTRODUCE YOURSELF, EXPLAIN THE PURPOSE OF THE SURVEY AND ASK THE FOLLOWING QUESTIONS. | | | | | | | | | | | | | | | | | | |
|  | I would like to know if the following medicines are available today in this facility. I would also like to observe the medicines that are available. If any of the medicines I mention is stored in another location in the facility, please tell me where in the facility it is stored so I can go there to verify. | | | | | | | | | | | | | | | | | | |
| R3 | 200 | Are any of the following medicines for the management of **hypertension** available in the facility today?  **CHECK TO SEE IF AT LEAST ONE OF EACH MEDICINE IS VALID (NOT EXPIRED)** | **OBSERVED AVAILABLE** | | | | **NOT OBSERVED** | | | | | | | | | | | |  |
|  |  |  | AT LEAST ONE VALID | | AVAILABLE NON VALID | | REPORTED AVAILABLE BUT NOT SEEN | | | NOT AVAILABLE TODAY | | | | | | NEVER AVAILABLE | | |  |
|  | 01 | Enalapril | 1 | | 2 | | 3 | | | 4 | | | | | | 5 | | |  |
|  | 02 | Enalapril+Hydrochlorothiazid | 1 | | 2 | | 3 | | | 4 | | | | | | 5 | | |  |
|  | 03 | Nifedipin | 1 | | 2 | | 3 | | | 4 | | | | | | 5 | | |  |
|  | 04 | Carvedilol | 1 | | 2 | | 3 | | | 4 | | | | | | 5 | | |  |
|  | 05 | Losartan |  | |  | |  | | |  | | | | | |  | | |  |
|  | 06 | Losartan + hydroclorothiazid | 1 | | 2 | | 3 | | | 4 | | | | | | 5 | | |  |
|  | 07 | Nicardipine | 1 | | 2 | | 3 | | | 4 | | | | | | 5 | | |  |
| **We have now completed all of the questions in this module of the survey.**  **Thank you for your participation.** | | | | | | | | | | | | | | | | | | |  |
| **SECTION 4: INTERVIEWER'S OBSERVATIONS** | | |  | | |  |  | | |  | | | | |  | | | |  |
| R4 | 100 | INTERVIEW END TIME (use the 24 hour-clock system) | \|___\| \|___\|: \|___\| \|___\| | | | | | | | | | | | |  | | | |  |
| R4 | 200 | RESULT CODES  (LAST VISIT): | COMPLETED ......................................................1 RESPONDENT NOT AVAILABLE ...................... 2 REFUSED ............................................................3 PARTIALLY COMPLETED .................................. 4 OTHER (SPECIFY)______________________ 96 | | | | | | | | | | | |  | | | |  |
|  |  | **COMMENTS ABOUT THE RESPONDENT:** | | | | | | | | | | | | |  | | | |  |
|  |  | ________________________________________________________________________________________________________________________________________________________________________________________________________________________________________________________________________________________________________ | | | | | | | | | | | | |  | | | |  |
|  |  | **COMMENTS ON SPECIFIC QUESTIONS:** | | | | | | | | | | | | |  | | | |  |
|  |  | ________________________________________________________________________________________________________________________________________________________________________________________________________________________________________________________________________________________________________ | | | | | | | | | | | | |  | | | |  |
|  |  | **ANY OTHER COMMENTS:** | | | | | | | | | | | | |  | | | |  |
|  |  | ________________________________________________________________________________________________________________________________________________________________________________________________________________________________________________________________________________________________________ | | | | | | | | | | | | |  | | | |  |
|  |  | **SUPERVISOR'S OBSERVATIONS:** | | | | | | | | | | | | |  | | | |  |
|  |  | ________________________________________________________________________________________________________________________________________________________________________________________________________________________________________________________________________________________________________ | | | | | | | | | | | | |  | | | |  |
|  |  | **NAME OF SUPERVISOR: ____________________________________** | | | | | | | | | | | | |  | | | |  |
|  |  | **DATE: ___________________** | | | | | | | | | | | | |  | | | |  |
