# Supplementary material for: Assessment of availability, readiness, and challenges for scaling-up hypertension management services at primary healthcare facilities, Central Highland region, Vietnam, 2020
Source: BMC Prim Care. 2023 Jul 1;24:138. doi: 10.1186/s12875-023-02092-8 (PMC10315019; doi:10.1186/s12875-023-02092-8)
Supplement: Supplementary file 2 — Additional file 2. [file 12875_2023_2092_MOESM2_ESM.docx]

**Part 2**

**INDEPTH INTERVIEW GUIDE**

Thank you very much for your willingness to participate in this research. In the next approximately one hour, I would like to ask for your opinions regarding different aspects of your health facility. Specifically, I would like to know the availability and the readiness of hypertension services that your facility offers and how to improve the delivery of hypertension management services at your facility. If you are uncomfortable answering any question, then don’t answer. Information from the interview will be used for research purpose only. Let us begin with some basic information:

| Date of interview (*dd/mm/yyyy*): | __/__/____ |
| --- | --- |
| Interview starts at: | ___h___m |
| Interview ends at: | ___h___m |
| Name of research’s staff: | ___________________________ |
| Code of interviewee: | ___________________________ |
| Sex of interviewee: | ___________________________ |
| Interviewee’s year of birth | ___________________________ |
| Province: | ____________________________ |

**I. GENERAL INFORMATION**

1. What is your current position? What are your main tasks in hypertension management program?
2. What are your other responsibilities?
3. How long have you worked in hypertension management program?
4. Before working at the facility, where did you work for? For how long? In which area?
5. What is your specialty? Where did you get your education?

**II. FACILITY INFORMATION**

1. Please tell me briefly about your facility:

[Probes]:

- - - How many people who are working at this facility and their tasks?
    - What services does your facility for clients?
    - What do you think about the services at the facility?

**III. CURRENT HYPERTENSION MANAGEMENT SERVICES**

1. Please tell me about the availability of current hypertension management services that your facility is offering:

[Probes]:

- - - What hypertension management services that your facility is offering?
    - How often are those services offered?
    - How many staff are working for hypertension management services in this facility?
    - What is their educational background?
    - How are their working experience?

1. Please tell me about the readiness of current hypertension management services that your facility is offering:

[Probes]:

- - - What types of basic amenities for hypertension management services does your facility have? (power, sanitation, communication management…)
    - What types of medical equipment for hypertension management services does your facility have? (blood pressure apparatus, …)
    - How does that equipment operate?
    - How are hypertension clients diagnosed and managed in the facility?
    - How well are the essential medicines for hypertension prepared in the facility?
    - How is the medicine disposal process?

1. Please tell me about clients/patients in hypertension service:

[Probes]:

- - - How many clients/patients are there in the hypertension service at present?
    - How often do they visit the facility?
    - What are their demographic characteristics? (living place, age, economic condition,)

**IV. THE IMPLEMENTATION OF HYPERTENSION MANAGEMENT PROGRAM**

1. Generally, how do you think about the implementation of hypertension management program in the facility?
   - - Is your facility well equipped?
     - What (staff, equipment, medicines) do you need more to provide better hypertension management services at your facility?
     - What are advantages and challenges?
     - What are reasons?
     - Please describe any barriers due to policies?
     - What kind of support does the facility have to implement the program?
2. What plan do you have to improve the implementation of hypertension management program in the facility?
